# Scleroderma-specific autoantibodies embedded in immune complexes mediate endothelial damage: an early event in the pathogenesis of systemic sclerosis

**DOI:** 10.1186/s13075-020-02360-3

**Published:** 2020-11-09

**Authors:** Elena Raschi, Daniela Privitera, Caterina Bodio, Paola Adele Lonati, Maria Orietta Borghi, Francesca Ingegnoli, Pier Luigi Meroni, Cecilia Beatrice Chighizola

**Affiliations:** 1 Experimental Laboratory of Immunological and Rheumatologic Researches, IRCCS Istituto Auxologico Italiano, Via Zucchi 18, Cusano Milanino, 20095 Milan, Italy; 2 Department of Clinical Sciences and Community Health, University of Milan, Via Festa del Perdono 7, 20122 Milan, Italy; 3 Division of Clinical Rheumatology, Research Center for Adult and Pediatric Rheumatic Diseases, ASST G. Pini, Piazza C Ferrari 1, 20122 Milan, Italy; 4 Allergology, Clinical Immunology and Rheumatology Unit, IRCCS Istituto Auxologico Italiano, Piazzale Brescia 20, 20149 Milan, Italy

**Keywords:** Systemic sclerosis, Autoantibodies, Immune complexes, Toll-like receptors, Endothelial cells, Fibrosis, Inflammation

## Abstract

**Background:**

Consistently with their diagnostic and prognostic value, autoantibodies specific for systemic sclerosis (SSc) embedded in immune complexes (ICs) elicited a pro-inflammatory and pro-fibrotic cascade in healthy skin fibroblasts, engaging Toll-like receptors (TLRs) via their nucleic acid components. The objective of this study was to investigate the pathogenicity of SSc-ICs in endothelial cells.

**Methods:**

ICs were purified from the sera of SSc patients bearing different autoantibody specificities (antibodies against DNA topoisomerase I, centromeric proteins, RNA polymerase, and Th/To), patients with systemic lupus erythematosus (SLE) and primary anti-phospholipid syndrome (PAPS), or healthy controls (NHS) using polyethylene glycol precipitation. Human umbilical vein endothelial cells (HUVECs) were incubated with ICs, positive and negative controls. mRNA levels of *endothelin-1 (et-1)*, *collagenIα1 (colIα1)*, *interferon (IFN)-α*, and *IFN-β* were investigated by real-time PCR; *et-1* and *il-6* mRNA levels were assessed after pre-treatment with bafilomycin. ICAM-1 expression was evaluated by cell ELISA; secretion of IL-6, IL-8, and transforming growth factor (TGF)-β1 in culture supernatants was measured by ELISA. The expression of Fcγ receptors (CD64, CD32, and CD16) was assessed in endothelial cells at FACS analysis. Intracellular signaling pathways culminating with NFκB, p38MAPK, SAPK-JNK, and Akt were assessed by Western blotting. Healthy skin fibroblasts were stimulated with supernatants from HUVECs incubated with ICs, and TGF-β1 secretion and mRNA levels of *colIα1* and *matrix metalloproteinase (mmp)-1*, protein expression of α smooth muscle actin (α-SMA), and IL-6 were evaluated by Western blotting; *et-1* mRNA levels were assessed in fibroblasts pre-treated with IL-6 and TGF-β inhibitors and stimulated with ATA-ICs.

**Results:**

All SSc stimulated IL-6 secretion; ACA-ICs and anti-Th/To-ICs increased ICAM-1 expression; all SSc-ICs but anti-Th/To-ICs augmented IL-8 levels; all SSc-ICs but ACA-ICs and ARA-ICs upregulated *et-1*, and all SSc-ICs but ARA-ICs affected TGF-β1 secretion. c*olIα1*, *IFN-α*, and *IFN-β* mRNA levels were not affected by any SSc-IC. FcγRII (CD32) and FcγRIII (CD16) were not detectable on HUVECs, while FcγRI (CD64) was minimally expressed. A differential modulation of *tlr* expression was observed: *tlr2*, *tlr3*, and *tlr4* were upregulated by ATA-ICs and ACA-ICs, while anti-Th/To-ICs resulted in *tlr9* upregulation. Pre-treatment with bafilomycin did not affect the upregulation of *et-1* and *il-6* induced by ATA-ICs, ACA-ICs, and anti-Th/To-ICs; a 23% reduction in both genes was reported for ARA-ICs. All SSc-ICs activated p38MAPK and Akt, and all SSc-ICs but ARA-ICs yielded the activation of NFκB; ATA-ICs and ACA-ICs increased the activation rate of both subunits of SAPK-JNK. When healthy skin fibroblasts were stimulated with supernatants from HUVECs incubated with SSc-ICs, TGF-β1 secretion, *colIα1*, α-SMA, and IL-6 expression levels were significantly modulated. Pre-treatment with IL-6 and TGF-β inhibitors prevented *et-1* upregulation induced by ATA-ICs by 85% and 77%, respectively.

**Conclusions:**

These data provide the first demonstration of the pathogenicity of ICs from scleroderma patients with different autoantibodies on the endothelium. Endothelial activation induced by SSc-ICs ultimately led to a pro-fibrotic phenotype in healthy skin fibroblasts.

## Background

Systemic sclerosis (SSc) is a chronic systemic autoimmune condition, burdened by the highest mortality among all the rheumatic diseases [1]. The prominent clinical feature of SSc is cutaneous involvement: fibrosis leads to skin tightness, itchiness, loss of skin appendages, and subcutaneous fat. It is thus not surprising that the disease was originally named “scleroderma,” from the ancient Greek words “σχλερος” (tight) and “δερμα” (skin) [2]. Over time, it has been progressively acknowledged that SSc might be highly polymorphic in terms of clinical presentations. Besides the skin, the fibrotic derangement with deposition of collagen and other components of extra-cellular matrix (ECM) might virtually affect any organ, culminating in tissue dysfunction: most commonly, the gastrointestinal tract, the heart, and the lungs. Other clinical manifestations of SSc acknowledge a vascular etiology: pulmonary arterial hypertension (PAH), scleroderma renal crisis (SRC), and gastric antral vascular ectasia (GAVE) are underpinned by fibroproliferative microangiopathy [3]. Given the complexity of the clinical picture, the major challenge in SSc consists of the accurate stratification of the risk of future complications. To date, the most reliable tool to predict the pattern of organ involvement is provided by the fine specificity of SSc-specific autoantibodies [4]. These autoantibodies are generally mutually exclusive and highly specific for SSc, being incorporated in the most recent classification criteria for this condition [5]. The autoantibody reactivity allows patients to be stratified early in the disease course, leading to a tailored approach and management [3, 5, 6]. Antibodies against DNA topoisomerase I (ATA) are predictors of the development of interstitial lung disease (ILD) and digital ulcers but appear to be protective against PAH. Antibodies against centromeric proteins (ACA) predict subcutaneous calcinosis, PAH, and gastrointestinal involvement but confer protection against the development of SRC, ILD, synovitis, tendon friction rubs, joint contractures, and myopathy. Antibodies directed against RNA polymerase III (ARA) have been linked to severe cutaneous involvement, with flexion contracture of the hands and tendon friction rubs. ARA provide one of the strongest risk factors for SRC and GAVE, being protective against ILD and inflammatory myopathy. Antibodies against Th/To (anti-Th/To) correlate with limited cutaneous involvement and severe ILD [4, 7].

The strong association with SSc and the role as prognostic biomarkers suggest the potential pathogenicity of these autoantibodies, similarly to what reported for systemic lupus erythematosus (SLE) [8], anti-phospholipid syndrome (APS) [9], Sjogren’s syndrome [10] and congenital cardiac block [11], inflammatory myopathies [12], and immune-mediated necrotizing myopathies [13]. Evidence of the pathogenic role of SSc-specific antibodies was raised by our group, with the demonstration that scleroderma-specific autoantibodies bound to their antigens to form immune complexes (ICs) elicit pro-inflammatory and pro-fibrotic effects on healthy skin fibroblasts. According to our previous work, the effects of SSc-ICs on fibroblasts might be mediated by Toll-like receptors (TLRs) interacting with the nucleic acid fragments embedded in scleroderma ICs [14].

Fibroblasts are regarded as master players in SSc pathogenesis, being responsible for the ultimate event, namely the excessive deposition of the extra-cellular matrix (ECM). However, the etiopathogenesis of such a complex disease is an intricate interplay between many cell types: besides fibroblasts, endothelial cells are key pathogenic effectors [15]. Therefore, the aim of this study was to broaden our previous findings on the pathogenicity of SSc-ICs by assessing the pro-inflammatory and pro-fibrotic effects of scleroderma ICs in healthy endothelial cells.

## Material and methods

### Serum samples

Serum samples were obtained from twelve patients with SSc fulfilling the 2013 ACR/EULAR criteria [5]. All patients had anti-nuclear antibodies (ANA) at indirect immunofluorescence on HEp-2 cells, at a titer greater than 1:160, with staining patterns consistent with the antigenic specificity. Four patients carried ATA, four ACA, two ARA, and two anti-Th/To. The remaining autoantibody profile was negative. In all cases, antibody reactivities against scleroderma antigens were confirmed using two different techniques: line blot (“EUROLINE-SSc profile,” Euroimmun, Lubeck, Germany, which includes the following antigens: Ro52, PDGF receptor, Ku, Pm/Scl75, PM-Scl100, Th/To, NOR90, fibrillarin, RP155, RNA polymerase III RP11, CENP B, CENP A, and DNA topoisomerase I) and chemiluminescent immunoassays (“QUANTA Flash CTD Screen Plus,” INOVA Diagnostics, San Diego, CA, USA, which detects antibodies against dsDNA, Sm/RNP, Ro52, Ro60, SSB, DNA topoisomerase I, centromere, Mi-2, Ku, Th/To, RNA polymerase III, Pm/Scl, PCNA, Jo-1, and ribosomal P). The demographic and clinical features of enrolled SSc patients are detailed in Table 1.

**Table 1:** Demographic and clinical data of enrolled SSc patients

	ATA (***n*** = 4)	ACA (***n*** = 4)	ARA (***n*** = 2)	Anti-Th/To (***n*** = 2)
**Age (years)**	51 [44.25–55.25]	51 [43.5–60]	37.5 [31.25–43.75]	41.5 [38.75–44.25]
**Females (%)**	4 (100%)	4 (100%)	2 (100%)	2 (100%)
Disease duration (months)*	31 [29.5–33.5]	30.5 [26–34.5]	28 [25–31]	31.5 [30.25–32.75]
**dcSSc/lcSSc°**	3/1	3/1	2/0	0/2
**ILD (%)**^**¶**^	2 (50%)	0	0	1 (50%)
**PAH (%)**^**§**^	0	1 (25%)	0	1 (50%)
**Joint involvement (%)**	1 (25%)	0	1 (50%)	0
**SRC (%)**^**#**^	0	0	0	0
**Muscle involvement (%)**^**$**^	0	0	0	1 (25%)
**Severe GI involvement (%)**^**&**^	0	1 (25%)	0	0
**Heart involvement (%)**^**£**^	1 (25%)	0	0	0
**Digital ulcers (%)**	1 (25%)	1 (25%)	0	0
**Raynaud’s phenomenon (%)**	4 (100%)	4 (100%)	2 (100%)	1 (50%)
**DMARDs**				
**HCQ**	1	2	1	1
**AZA**	0	0	0	1
**MTX**	2	0	1	0
**MMF**	1	0	0	0

Continuous variables are expressed as median [interquartile range]

*dcSSc* diffuse cutaneous systemic sclerosis, *lcSSc* limited cutaneous systemic sclerosis, *RP* primary Raynaud’s phenomenon, *n* number, *ACA* antibodies against centromeric proteins, *ATA* antibodies against DNA topoisomerase I, *ARA* antibodies against RNA polymerase III, *anti-Th/To* antibodies against Th/To, *ILD* interstitial lung disease, *SRC* scleroderma renal crisis, *GI* gastrointestinal, *DMARDs* disease-modifying anti-rheumatic drugs, *HCQ* hydroxychloroquine, *AZA* azathioprine, *MTX* methotrexate, *MMF* mycophenolate

*From the onset of the first non-Raynaud’s phenomenon symptom to study inclusion

^¶^Forced vital capacity (FVC) or carbon monoxide diffusing capacity of the lung (DL_CO_) < 55% of predicted or a 15% decline from baseline in FVC or DL_CO_, with evidence of fibrosis on high-resolution CT

^§^Mean pulmonary arterial pressure > 25 mmHg at right heart catheterization

^#^New-onset systemic hypertension > 150/85 mmHg with a decrease in the estimated glomerular filtration rate > 30%

^$^Objective muscle weakness (score < 4 on a 5-point Likert scale) and an elevated total creatine kinase level (> 4-fold the upper limit of normal)

^&^At least 3 episodes of intestinal pseudoobstruction requiring hospitalization or requiring > 6 weeks of enteral or parental nutritional support

^£^Hemodynamically significant arrhythmias, pericardial effusion, or congestive heart failure

°According to LeRoy [16]

Two SLE patients were recruited; one patient carried anti-Sm, anti-U1 ribonucleoprotein (RNP), and anti-double-stranded DNA antibodies, the other harbored anti-Sm [17]. Serum was also obtained from two subjects with primary anti-phospholipid syndrome (PAPS) and positive lupus anticoagulant test, anti-cardiolipin, and anti-β2 glycoprotein I IgG antibodies [18]. Four normal healthy subjects (NHS), matched on age and gender to patients, with no autoimmune disease and negative autoantibody profile, were enrolled. Serum samples were stored at − 20 °C.

### Endothelial cell culture

Human umbilical vein endothelial cells (HUVECs) were isolated from normal-term umbilical cord vein by type II collagenase perfusion (Worthington, Lakewood, NJ, USA). HUVEC cultures were maintained in complete E199 medium (Flow Labs) supplemented with 20% heat-inactivated fetal bovine serum (FBS; PAA Laboratories-GE Healthcare), 1% l-glutamine (MP Biomedicals Inc.), 100 U/ml penicillin-streptomycin (MP Biomedicals), and 250 ng/ml Amphotericin B (MP Biomedicals). Confluent cells were passaged with a 0.25% trypsin/EDTA (Gibco-Life Technologies) [19]. In all the experiments, a pool of cells from at least three donors was used at the first passage.

### Healthy skin fibroblast cell culture

Dermal fibroblasts were isolated from skin biopsies from two NHS. Under local anesthesia with 1% xylocaine, 5-mm punch skin biopsies were performed in the distal forearm. Samples were minced into small pieces and digested by collagenase type I (Thermo Fisher Scientific Inc., Waltham, MA, USA) for 2 h at 37 °C with 5% CO_2_. After centrifugation at 300*g* for 10 min, pellets were resuspended in 1 ml D-MEM (Gibco-Life Technologies, Groningen, The Netherlands) supplemented with 20% fetal bovine serum (FBS, PAA-GE Healthcare, Buckinghamshire, UK), 2 mM glutamine (Sigma-Aldrich), and penicillin (100 U/ml)-streptomycin (100 μg/ml) (Sigma-Aldrich) and transferred into a T25 plate (Corning Incorporated, NY, USA). Cultures were maintained at 37 °C in 5% CO_2_-humidified incubator until confluence. Non-adherent cells and dermal tissue were removed by washing; established fibroblasts were passaged after trypsin/EDTA (Thermo Fisher Scientific) release up to the eight passage. Cells were maintained in D-MEM with 10% FBS, 2 mM glutamine, and penicillin (100 U/ml)-streptomycin (100 μg/ml) (Thermo Fisher Scientific) or incubated overnight in D-MEM with 1% FBS before functional studies. The purity of fibroblast culture was 98% as detected by flow cytometry using a mouse anti-human CD90 and a mouse anti-human CD45 antibodies–PE-conjugated (BD Biosciences, San Jose, CA, USA).

Healthy skin fibroblasts were stimulated with supernatants from HUVECs incubated with scleroderma and control ICs for 24 h. The mRNA levels of collagen *(col)Iα1* and matrix metalloproteinases *(mmp)-1*, the secretion of transforming growth factor (TGF)-β1, and the protein expression levels of α smooth muscle actin (α-SMA) and IL-6 were evaluated. In the latter experiments, fibroblasts were also stimulated with tumor necrosis factor (TNF)-α (10 ng/ml, R&D Systems) and with supernatants from HUVECs incubated with TNF-α for 24 h as positive controls.

### Immune complexes

ICs were precipitated from NHS’ and patients’ sera. Briefly, serum samples were mixed with ice-cold 5% polyethylene glycol (PEG) 6000 (Sigma-Aldrich; Saint Louis, MO, USA)-0.1 M EDTA (Bioscience, Inc., La Jolla, CA, USA) and incubated overnight at 4 °C. Samples were diluted three times with 2.5% PEG 6000 in RPMI (Euroclone S.p.A., Pero, Italy), layered on top of 2.5% PEG 6000 supplemented with 5% human serum albumin (Sigma-Aldrich), and centrifuged at 3900 rpm at 4 °C for 20 min. Pellets were dissolved in D-PBS to the initial serum volume and immediately used at 1:2 dilution [20]. Every sample was used in triplicates, and each experiment was repeated twice using SSc-ICs isolated from all patients for each autoantibody specificity and control ICs.

The potential endotoxin contamination of IC preparations was ruled out by limulus amoebocyte lysate (LAL) gel-clot test (Pyrosate Kit, Associates of Cape Cod Incorporated, East Falmouth, MA, USA; sensitivity 0.25 EU/ml).

### ICAM-1 expression

Inter-cellular adhesion molecule (ICAM)-1 surface levels were evaluated by home-made cell ELISA, as in previous studies [21]. Confluent HUVEC monolayers were rested in D-MEM with 1% FBS overnight in a 96-well plate. After 24-h incubation with 100 μl/well of SSc-ICs, PAPS-ICs, SLE-ICs, NHS-ICs, IL-1β (50 U/ml, PeproTech, Rocky Hill, NJ, USA), LPS (1 μg/ml, R&D Systems, Minneapolis, MN, USA), or medium alone, cells were washed twice with HBSS (Sigma-Aldrich) and incubated for 60 min at room temperature with 100 μl/well of murine monoclonal IgG specific for human ICAM-1 (CD54, R&D Systems). The antibody was used at a final dilution of 1:500 in HBSS-FBS 2.5%. After two additional washes, cells were incubated for 90 min at room temperature with 100 μl of phosphatase-conjugated goat anti-mouse IgG (Cappel, Cochranville, PA, USA). The secondary antibody was used at a dilution of 1:1000 in HBSS-FBS 10%. After two washes with HBSS, 100 μl of the enzymatic substrate (p-nitrophenylphosphate in 0.05 M Mg-carbonate buffer pH 9.8, Sigma-Aldrich) was added. The optical density (OD) values were evaluated at 405 nm after 30 min of incubation by a semiautomatic reader (Titertek Multiskan MCC/340, Titertek Instruments Inc., Pforzheim, Germany).

### IL-6, IL-8, and TGF-β1 protein secretion

IL-6, IL-8, and TGF-β1 release was evaluated in culture supernatants after 24-h incubation with SSc-ICs, PAPS-ICs, SLE-ICs, NHS-ICs, or agonists [IL-1β and LPS] by commercial ELISAs (R&D Systems).

### Fcγ receptor expression

The expression of FcγRI (CD64), FcγRII (CD32), and FcγRIII (CD16) was measured on HUVECs by flow cytometry after subtraction of background signals. Briefly, cells were detached by trypsin/EDTA and washed; 200,000 cells per tube were incubated for 20 min at 4 °C with FITC mouse monoclonal anti-human CD64 (Thermo Fisher), FITC mouse monoclonal anti-human CD32 (Beckman Coulter, Brea, CA, USA), FITC mouse monoclonal anti-human CD16 (BD Pharmingen, San Diego, CA, USA), or mouse anti-human isotype control (BD Biosciences, San Jose, CA). Samples were washed again and suspended in 250 μl cold DPBS/1% FCS. Ten thousand events were acquired at a medium flow rate. A single fluorochrome dot plot strategy was used to identify FcγR-positive endothelial cells. In the experiments, control procedures to establish proper calibration and linearity were performed. Analyses were performed using the BD FACSLyric cytometer and BD FACSuite software (BD Biosciences).

### tlr2, tlr3, tlr4, tlr7, tlr8, tlr9, interferon-α, interferon-β, endothelin-1, matrix metalloproteinase-1, and collagenIα1 mRNA expression levels

*tlr2*, *tlr3*, *tlr4*, *tlr7*, *tlr8*, *tlr9*, *interferon (ifn)-α*, *ifn-β*, and *endothelin (et)-1* mRNA expression levels were evaluated in HUVECs stimulated for 24 h with SSc-ICs, PAPS-ICs, SLE-ICs, NHS-ICs, or agonists (LPS, Poly I:C, and ODN CpG [5 μM, InvivoGen, San Diego, CA, USA]). *mmp-1* and *colIα1* were evaluated in healthy skin fibroblasts stimulated for 24 h with supernatants from HUVECs treated with SSc-ICs, NHS-ICs, or recombinant human TGF-β1 (10 ng/ml, PreproTech, Rocky Hill, NJ, USA). Total RNA from cells was purified using TRIzol Reagent (Thermo Fisher Scientific). Amplification Grade DNase I (Thermo Fisher Scientific) was used to eliminate residual genomic DNA. A reverse transcription reaction was performed using the SuperScript™ First-Strand Synthesis System for RT-PCR (Thermo Fisher Scientific). Universal PCR Master Mix No AmpErase UNG (Thermo Fisher Scientific) was used for quantitative RT-PCR, by ABI PRISM 7900 HT Sequence Detection System (Thermo Fisher Scientific). Quantification of mRNA expression was performed with TaqMan® Gene Expression Assay (Thermo Fisher Scientific) for each target gene. The following TaqMan® Gene Expression assays were used: Hs01872448_s1 (*tlr2)*, Hs01551078_m1 (*tlr3*), Hs00152939_m1 (*tlr4*), Hs01933259_s1 (*tlr7*), Hs00152972_m1 (*tlr8*), Hs00370913_s1 (*tlr9*), Hs00855471_g1 (*ifn-α*), Hs01077958_s1 (*ifn-β*), Hs00174961_m1 (*et-1*), Hs00164004_m1 (*colIα1*), Hs00899658_m1 (*mmp-1*), and Hs99999905_m1 (*gapdh*). Expression levels of target genes (*tlr2*, *tlr3*, *tlr4*, *tlr7 tlr8* and *tlr9*, *ifn-α*, *ifn-β*, *et-1*, *mmp-1*, and *coIα1*) were determined by the comparative Ct method normalizing the target to the endogenous gene (*gapdh*). Relative values of target to reference were expressed as fold change (RQ). The optimal time point to evaluate the mRNA levels of *colIα1* was set at 24 h based on a kinetics curve of the mRNA response to stimulation with TGF-β.

### IL-6 and α-SMA protein expression and nuclear factor κ B, p38 mitogen-activated kinase, SAPK-JNK, and Akt activation rate

IL-6 and α-SMA protein expression was evaluated in fibroblasts stimulated for 24 h with TNF-α and supernatants from HUVECs treated with SSc-ICs, NHS-ICs, and TNF-α. The activation rate of nuclear factor κ B (NFκB), p38 mitogen-activated kinase (p38MAPK), SAPK-JUN N terminal kinase (JNK), and RAC-α serine/threonine-protein kinase (Akt) was assessed in HUVECs incubated for 24 h with SSc-ICs, PAPS-ICs, SLE-ICs, NHS-ICs, and IL-1β. Total proteins were isolated using RIPA Lysis Buffer added with Protease and Phosphatase Inhibitor Cocktail (Sigma-Aldrich). Protein concentration was evaluated using the BCA Protein Assay Kit (Thermo Fisher Scientific). Proteins were fractionated by NuPAGE BIS-TRIS by 4–12% SDS-polyacrylamide pre-cast gel electrophoresis and transferred to nitrocellulose using iBlot Transfer Stacks Nitrocellulose (Thermo Fisher Scientific). The membranes were blocked for 2 h at room temperature in PBS/0.05% Tween 20 (PT) (Bio-Rad Laboratories, Hercules, CA, USA) containing 5% non-fat milk powder (Mellin, Milan, Italy) and incubated with anti-human IL-6 (Cell Signaling Technology, Danvers, MA, USA), anti-α smooth muscle actin (α-SMA, Sigma-Aldrich), anti-human α-tubulin (Sigma-Aldrich), anti-human NFκB, anti-human phosphorylated NFκB (pNFκB), anti-human p38MAPK, anti-human phosphorylated p38MAPK (pp38MAPK), anti-human SAPK-JNK or anti-human phosphorylated SAPK-JNK (anti-pSAPK-JNK) antibodies, anti-human Akt, or anti-human phosphorylated Akt (anti-pAkt, Cell Signaling Technology, Danvers, MA, USA). After washes, the membranes were incubated in PT/5% non-fat milk powder plus HRP-conjugated secondary antibodies (MP Biomedicals, Santa Ana, CA, USA) and developed using the ECL Plus Detection System (Thermo Fisher Scientific). Signals were detected using radiographic films (Kodak, Rochester, NY, USA). The ImageJ software (LI-COR Biosciences, Lincoln, NE, US) was used to analyze and quantify gels. Protein expression levels of IL-6 and α-SMA were normalized to the housekeeping gene, α-tubulin, and expressed as relative protein levels.

Activation kinetics to evaluate the phosphorylation of intracellular mediators in response to different ICs has been performed at different time points: 0–6, 24, 36 h. At 6 h, the activation rate of all intracellular study mediators was low. In all cases, the highest activation levels were observed at 24 and 36 h. However, we observed a decrease in cell viability at 36 h. Therefore, cells were incubated for 24 h with different stimuli.

### Bafilomycin pre-treatment

HUVECs were incubated for 2 h at 37 °C with bafilomycin A1 (Millipore, Temecula, CA, USA, 100 nmol) and then treated with SSc-ICs, PAPS-ICs, NHS-ICs, and Poly I:C. RT-PCR for *il-6* and *et-1* was then performed.

### IL-6 and TGF-β inhibitors

Fibroblasts were pre-incubated for 30 min at 37 °C with inhibitors of IL-6 (rat anti-human monoclonal antibody, Thermo Fisher Scientific Inc.; 2.5 μg/ml) and TGF-β (mouse anti-human monoclonal antibody, R&D Systems; 5 μg/ml). Pre-treated fibroblasts were then stimulated with supernatants from HUVECs incubated with ATA-ICs and with recombinant TGF-β1 as a positive control. The mRNA levels of *et-1* were evaluated.

### Statistical analysis

Descriptive statistics was used to calculate the mean and standard deviation (SD). Since our data were derived from in vitro experiments conducted under highly controlled conditions and originated from a high number of cells, the ANOVA test was used to compare different experimental conditions, and post hoc comparisons were assessed by Dunnett’s test. With regard to not homogeneity of variance assumption, Welch’s correction was applied when required. Paired or unpaired *t* tests were performed to compare the mean values between two groups. All analyses were performed with GraphPad Prism 5.01. *p* values < 0.05 were considered significant.

The approval of the Institutional Review Board of Istituto G. Pini, Milan, Italy, was obtained; all subjects provided written informed consent.

## Results

### ICAM-1 expression in endothelial cells treated with immune complexes

**Fig. 1:**
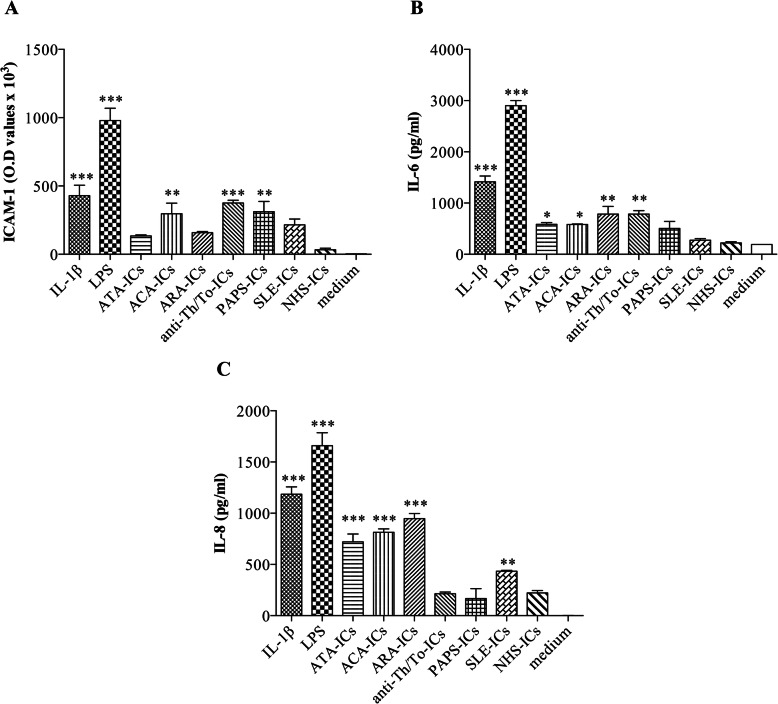
ICAM-1 expression and IL-6 and IL-8 secretion in HUVECs stimulated with SSc-ICs, PAPS-ICs, SLE-ICs, or NHS-ICs. Endothelial cells were incubated with SSc-ICs, PAPS-ICs, SLE-ICs, or NHS-ICs (1:2 dilution). IL-1β (50 U/ml) and LPS (1 μg/ml) were used as positive controls. Histograms represent mean + standard error of the mean (SEM). *p < 0.01; **p < 0.001; ***p < 0.0001 versus medium. **a** ICAM-1 expression (O.D.) [mean ± SD]. IL-1β [429.8 ± 151.9] versus medium [2.75 ± 1.71] (*p* < 0.0001) versus NHS-ICs (*p* < 0.0001). LPS [979.5 ± 180.3] versus medium (*p* < 0.0001) versus NHS-ICs (*p* < 0.0001). ATA-ICs [134 ± 16.27] versus medium (*p* = N.S.) and versus NHS-ICs [34.25 ± 17.63] (*p* = N.S.). ACA-ICs [297.3 ± 153.7] versus medium (*p* < 0.001) versus NHS-ICs (*p* < 0.01). ARA-ICs [157.8 ± 17.61] versus medium (*p* = N.S.) versus NHS-ICs (*p* = N.S.). Anti-Th/To-ICs [375 ± 42.16] versus medium (*p* < 0.0001) versus NHS-ICs (*p* < 0.0001). PAPS-ICs [311.3 ± 151.7] versus medium (*p* < 0.001) versus NHS-ICs (*p* < 0.001). SLE-ICs [216 ± 84.75] versus medium (*p* = N.S.) versus NHS-ICs (*p* = N.S.). NHS-ICs [34.25 ± 17.63] versus medium (*p* = N.S.). **b** IL-6 expression (pg/ml) [mean ± SD]. IL-1β [1414 ± 161.2] versus medium [191 ± 0.70] (*p* < 0.0001) versus NHS-ICs (*p* < 0.0001). LPS [2900.5 ± 141.4] versus medium (*p* < 0.0001) versus NHS-ICs (*p* < 0.0001). ATA-ICs [584.5 ± 48.79] versus medium (*p* < 0.01) and versus NHS-ICs [222.5 ± 31.82] (*p* = N.S.). ACA-ICs [582 ± 15.56] versus medium (*p* < 0.01) versus NHS-ICs (*p* = N.S). ARA-ICs [785.5 ± 210] versus medium (*p* < 0.001) versus NHS-ICs (*p* < 0.001). Anti-Th/To-ICs [786 ± 90.51] versus medium (*p* < 0.001) versus NHS-ICs (*p* < 0.001). PAPS-ICs [504.5 ± 193] versus medium (*p* = N.S.) versus NHS-ICs (*p* = N.S.). SLE-ICs [276.5 ± 40.31] versus medium (*p* = N.S.) versus NHS-ICs (*p* = N.S.). NHS-ICs [222.5 ± 31.82] versus medium (*p* = N.S.). **c** IL-8 expression (pg/ml) [mean ± SD]. IL-1β [1186 ± 101.8] versus medium [1.5 ± 0.6] (*p* < 0.0001) versus NHS-ICs (*p* < 0.0001). LPS [1659 ± 178.2] versus medium (*p* < 0.0001) versus NHS-ICs (*p* < 0.0001). ATA-ICs [721.5 ± 108.2] versus medium (*p* < 0.0001) and versus NHS-ICs [222.5 ± 31.82] (*p* < 0.001). ACA-ICs [814.5 ± 47.38] versus medium (*p* < 0.0001) versus NHS-ICs (*p* < 0.0001). ARA-ICs [948 ± 69.3] versus medium (*p* < 0.0001) versus NHS-ICs (*p* < 0.0001). Anti-Th/To-ICs [214 ± 22.63] versus medium (*p* = N.S) versus NHS-ICs (*p* = N.S.). PAPS-ICs [167 ± 137.2] versus medium (*p* = N.S.) versus NHS-ICs (*p* = N.S.). SLE-ICs [434 ± 11.31] versus medium (*p* < 0.001) versus NHS-ICs (*p* = N.S.). NHS-ICs [218.2 ± 28.51] versus medium (*p* = N.S)

ACA-ICs, anti-Th/To-ICs, and PAPS-ICs significantly induced ICAM-1 expression on HUVEC monolayers compared to medium; conversely, no increase in ICAM-1 expression was observed with ATA-ICs, ARA-ICs, and SLE-ICs. Both IL-1β and LPS elicited a significant increase in ICAM-1 protein levels compared to medium (Fig. 1a).

### IL-6 and IL-8 secretion in endothelial cells treated with immune complexes

All SSc-ICs enhanced IL-6 levels compared to the medium. Similarly, IL-1β and LPS drove a significant increase in IL-6 with respect to the medium. Conversely, HUVECs incubated with PAPS-ICs, SLE-ICs, and NHS-ICs exhibited IL-6 levels similar to cells treated with medium alone (Fig. 1b). All SSc-ICs but anti-Th/To-ICs augmented IL-8 levels compared to the medium. IL-1β, LPS, and SLE-ICs upregulated IL-8, whereas PAPS-ICs and NHS-ICs did not affect IL-8 levels (Fig. 1c).

### *et-1* and *ifn* mRNA expression in endothelial cells treated with immune complexes

**Fig. 2:**
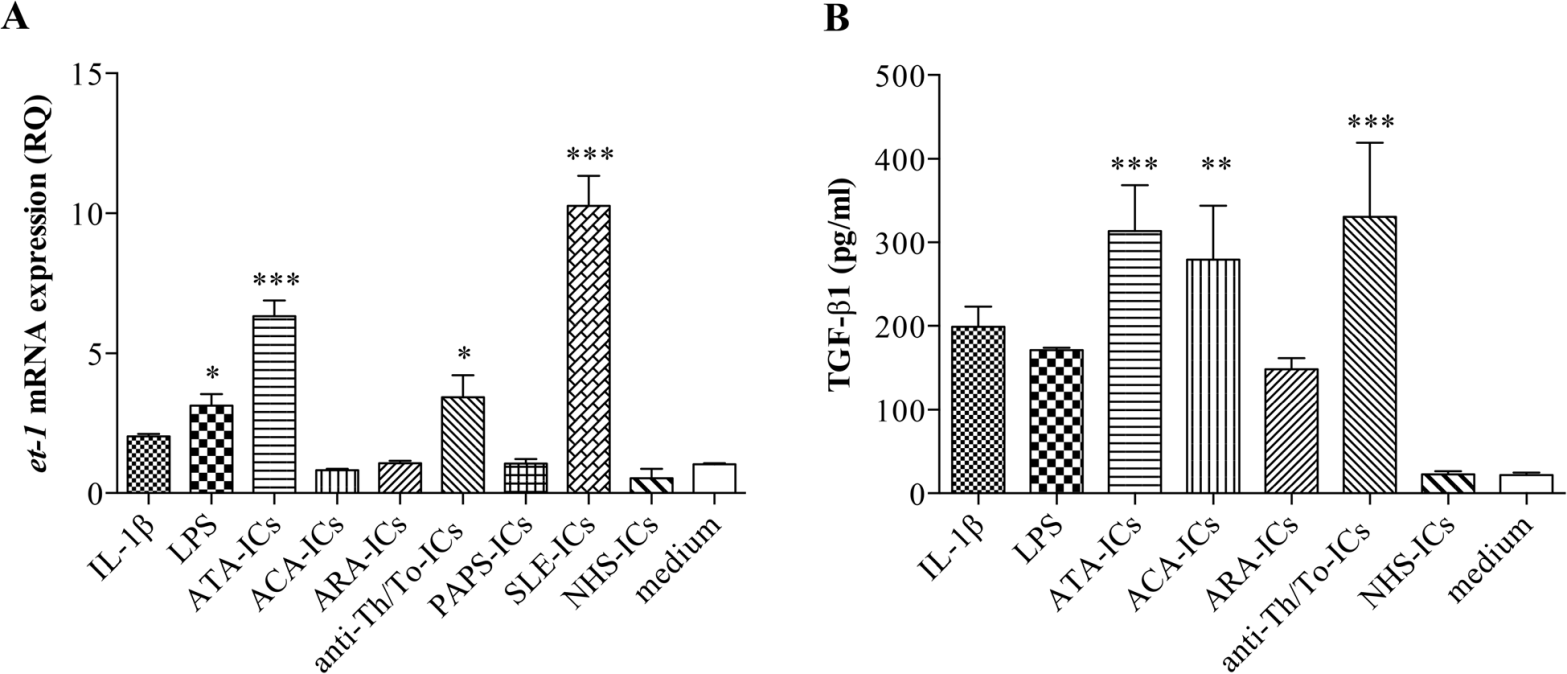
*et-1* mRNA expression levels in HUVECs stimulated with SSc-ICs, PAPS-ICs, SLE-ICs, and NHS-ICs; TGF-β1 secretion in HUVECs stimulated with SSc-ICs or NHS-ICs. Endothelial cells were incubated with SSc-ICs, PAPS-ICs, SLE-ICs, or NHS-ICs (1:2 dilution). IL-1β (50 U/ml) and LPS (1 μg/ml) were used as positive controls. *p < 0.01; **p < 0.001; ***p < 0.0001 versus medium. **a** *et-1* (RQ) [mean ± SD]. IL-1β [2.03 ± 0.15] versus medium [1.03 ± 0.06] (*p* = N.S.) versus NHS-ICs (*p* = N.S.). LPS [3.13 ± 0.71] versus medium (*p* < 0.01) versus NHS-ICs (*p* < 0.001). ATA-ICs [6.32 ± 0.99] versus medium (*p* < 0.0001) and versus NHS-ICs [0.53 ± 0.59] (*p* < 0.0001). ACA-ICs [0.80 ± 0.10] versus medium (*p* = N.S.) versus NHS-ICs (*p* = N.S.). ARA-ICs [1.07 ± 0.15] versus medium (*p* = N.S.) versus NHS-ICs (*p* = N.S.). Anti-Th/To-ICs [3.43 ± 1.37] versus medium (*p* < 0.01) versus NHS-ICs (*p* < 0.001). PAPS-ICs [1.06 ± 0.27] versus medium (*p* = N.S.) versus NHS-ICs (*p* = N.S.). SLE-ICs [10.27 ± 1.86] versus medium (*p* < 0.0001) versus NHS-ICs (*p* < 0.0001). NHS-ICs [0.53 ± 0.59] versus medium (*p* = N.S.). **b** TGF-β1 (pg/ml) [mean ± SD]. IL-1β [198.8 ± 48.71] versus medium [21.75 ± 6.24] (*p* = N.S.) versus NHS-ICs (*p* = N.S.). LPS [171.5 ± 5.8] versus medium (*p* = N.S.) versus NHS-ICs (*p* = N.S.). ATA-ICs [313.5 ± 109.9] versus medium (*p* < 0.0001) and versus NHS-ICs [22.75 ± 7.59] (*p* < 0.0001). ACA-ICs [279.5 ± 129.0] versus medium (*p* < 0.001) versus NHS-ICs (*p* < 0.001). ARA-ICs [148.5 ± 26.71] versus medium (*p* = N.S.) versus NHS-ICs (*p* = N.S.). Anti-Th/To-ICs [330.8 ± 177.4] versus medium (*p* < 0.0001) versus NHS-ICs (*p* < 0.0001). NHS-ICs [22.75 ± 7.59] versus medium (*p* = N.S)

ATA-ICs and anti-Th/To-ICs yielded a significant upregulation of *et-1* levels compared to the medium, as well as LPS and SLE-ICs. Conversely, ACA-ICs, ARA-ICs, PAPS-ICs, NHS-ICs, and IL-1β did not exert any effect (Fig. 2a). Stimulation with IL-1β, LPS, SSc-ICs, and control ICs did not result in a significant modulation of mRNA levels of *ifn-α* and *ifn-β* compared to the culture medium.

### TGF-β1 secretion and *colIα1* mRNA expression in endothelial cells treated with immune complexes

All SSc-ICs but ARA-ICs evoked a significant upregulation of TGF-β1 secretion compared to medium alone, while NHS-ICs did not exert any effect. Both IL-1β and LPS did not significantly modulate TGF-β1 levels (Fig. 2b). The mRNA expression of *colIα1* was not significantly affected by stimulation with SSc-ICs, control ICs, or IL-1β.

### Modulation of *et-1* and *il-6* mRNA levels in endothelial cells treated with bafilomycin and immune complexes

Pre-treatment with bafilomycin modulated the expression of study mediators in response to stimulation with ARA-ICs. In particular, we observed a 23% reduction of both *et-1* and *il-6* mRNA levels. Bafilomycin did not affect the upregulation of *et-1* and *il-6* by ATA-ICs, ACA-ICs, anti-Th/To-ICs, and control ICs. Conversely, upon stimulation with Poly I:C, a 43% and 58% decrease in the expression of *et-1* and *il-6*, respectively, was reported after bafilomycin pre-treatment.

### Fcγ receptor expression in endothelial cells

At flow-cytometry analysis, FcγRII (CD32) and FcγRIII (CD16) were not detectable on HUVECs (approximately 2%). Minimal expression of FcγRI (CD64) was observed (approximately 9%) (data not shown).

### *tlr* mRNA expression in endothelial cells treated with immune complexes

**Fig. 3:**
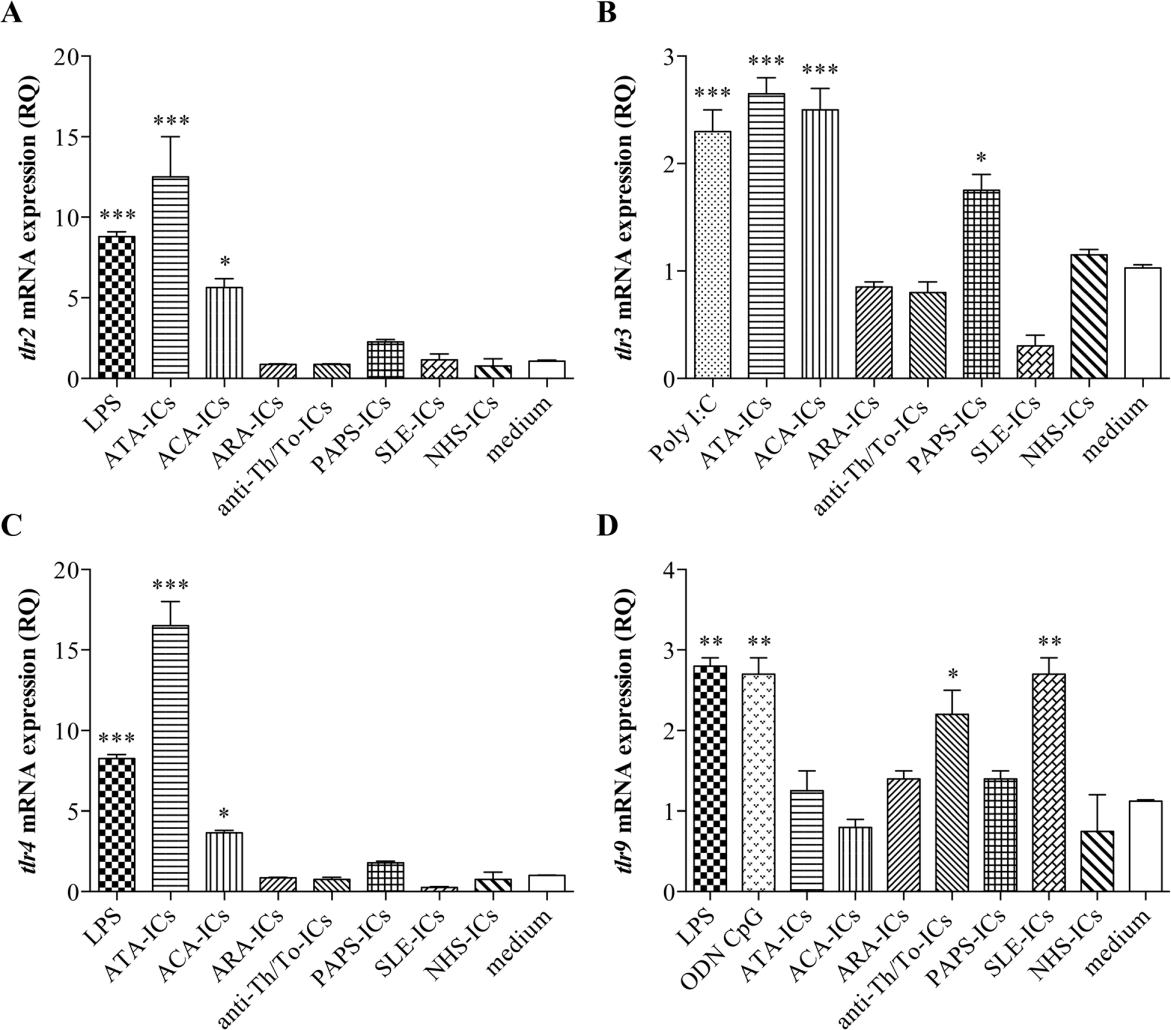
*tlr* mRNA expression levels in HUVECs stimulated with SSc-ICs, PAPS-ICs, SLE-ICs, and NHS-ICs. Endothelial cells were incubated with SSc-ICs, PAPS-ICs, SLE-ICs, or NHS-ICs (1:2 dilution). LPS (1 μg/ml), Poly I:C (1 μg/ml) and ODN CpG (5 μM) were used as positive controls. *p < 0.01; **p < 0.001; ***p < 0.0001 versus medium. **a** *tlr2* (RQ) [mean ± SD]. LPS [8.8 ± 0.42] versus medium [1.06 ± 0.08] (*p* < 0.0001) versus NHS-ICs (*p* < 0.0001). ATA-ICs [12.50 ± 3.54] versus medium (*p* < 0.0001) and versus NHS-ICs [0.75 ± 0.63] (*p* < 0.0001). ACA-ICs [5.65 ± 0.78] versus medium (*p* < 0.01) versus NHS-ICs (*p* < 0.01). ARA-ICs [0.83 ± 0.09] versus medium (*p* = N.S.) versus NHS-ICs (*p* = N.S.). Anti-Th/To-ICs [0.85 ± 0.23] versus medium (*p* = N.S.) versus NHS-ICs (*p* = N.S.). PAPS-ICs [2.25 ± 0.21] versus medium (*p* = N.S.) versus NHS-ICs (*p* = N.S.). SLE-ICs [1.15 ± 0.49] versus medium (*p* = N.S.) versus NHS-ICs (*p* = N.S.). NHS-ICs [0.75 ± 0.63] versus medium (*p* = N.S.). **b** *tlr3* (RQ) [mean ± SD]. Poly I:C [2.30 ± 0.28] versus medium [1.03 ± 0.04] (*p* < 0.0001) versus NHS-ICs (*p* < 0.0001). ATA-ICs [2.65 ± 0.21] versus medium (*p* < 0.0001) and versus NHS-ICs [1.15 ± 0.07] (*p* < 0.0001). ACA-ICs [2.5 ± 0.28] versus medium (*p* < 0.0001) versus NHS-ICs (*p* < 0.0001). ARA-ICs [0.85 ± 0.07] versus medium (*p* = N.S.) versus NHS-ICs (*p* = N.S.). Anti-Th/To-ICs [0.80 ± 1.14] versus medium (*p* = N.S.) versus NHS-ICs (*p* = N.S.). PAPS-ICs [1.75 ± 0.21] versus medium (*p* < 0.01) versus NHS-ICs (*p* < 0.01). SLE-ICs [0.30 ± 0.14] versus medium (*p* < 0.01) versus NHS-ICs (*p* < 0.001). NHS-ICs [1.15 ± 0.07] versus medium (*p* = N.S.). **c** *tlr4* (RQ) [mean ± SD]. LPS [8.25 ± 0.35] versus medium [1.01 ± 0.02] (*p* < 0.0001) versus NHS-ICs (*p* < 0.0001). ATA-ICs [16.50 ± 2.12] versus medium (*p* < 0.0001) and versus NHS-ICs [1.15 ± 0.07] (*p* < 0.0001). ACA-ICs [3.65 ± 0.21] versus medium (*p* < 0.01) versus NHS-ICs (*p* < 0.0001). ARA-ICs [0.80 ± 0.14] versus medium (*p* = N.S.) versus NHS-ICs (*p* < 0.01). Anti-Th/To-ICs [0.70 ± 0.28] versus medium (*p* = N.S.) versus NHS-ICs (*p* = N.S.). PAPS-ICs [1.85 ± 0.21] versus medium (*p* = N.S.) versus NHS-ICs (*p* = N.S.). SLE-ICs [0.35 ± 0.17] versus medium (*p* = N.S.) versus NHS-ICs (*p* = N.S.). NHS-ICs [1.21 ± 0.57] versus medium (*p* = N.S.). **d** *tlr9* (RQ) [mean ± SD]. LPS [2.8 ± 0.14] versus medium [1.12 ± 0.03] (*p* < 0.001) versus NHS-ICs (*p* < 0.0001). ODNCpG [2.7 ± 0.28] versus medium (*p* < 0.001) versus NHS-ICs (*p* < 0.0001). ATA-ICs [1.25 ± 0.35] versus medium (*p* = N.S.) and versus NHS-ICs [0.75 ± 0.64] (*p* = N.S.). ACA-ICs [0.9 ± 0.2] versus medium (*p* = N.S.) versus NHS-ICs (*p* = N.S.). ARA-ICs [1.4 ± 0.13] versus medium (*p* = N.S.) versus NHS-ICs (*p* = N.S.). Anti-Th/To-ICs [2.20 ± 0.42] versus medium (*p* < 0.01) versus NHS-ICs (*p* < 0.001). PAPS-ICs [1.4 ± 0.12] versus medium (*p* = N.S.) versus NHS-ICs (*p* = N.S.). SLE-ICs [2.70 ± 0.28] versus medium (*p* < 0.001) versus NHS-ICs (*p* < 0.0001). NHS-ICs [0.85 ± 0.49] versus medium (*p* = N.S)

ATA-ICs and ACA-ICs, as well as LPS, drove a significant increase in *tlr2* mRNA as compared to the medium. ARA-ICs, anti-Th/To-ICS, PAPS-ICs, SLE-ICs, and NHS-ICs did not significantly modulate *tlr2* mRNA (Fig. 3a). Similarly, ATA-ICs and ACA-ICs, but not ARA-ICs and anti-Th/To-ICs, promoted a significant *tlr3* upregulation; an increase in *tlr3* mRNA was also observed with Poly I:C and PAPS-ICs. SLE-ICs and NHS-ICs did not affect the mRNA levels of *tlr3* (Fig. 3b). ATA-ICs and ACA-ICs, as well as LPS, elicited a significant raise in *tlr4* mRNA levels. ARA-ICs and anti-Th/To-ICs, together with PAPS-ICs, SLE-ICs, and NHS-ICs, did not affect *tlr4* mRNA levels (Fig. 3c). *tlr9* expression was significantly modulated by anti-Th/To-ICs, SLE-ICs, LPS, and ODN CpG. Conversely, ATA-ICs, ARA-ICs, ACA-ICs, PAPS-ICs, and NHS-ICs did not affect *tlr9* mRNA levels (Fig. 3d). *tlr7* and *tlr8* mRNA could not be detected in HUVECs.

### Intra-cellular signaling pathways in endothelial cells treated with immune complexes

**Fig. 4:**
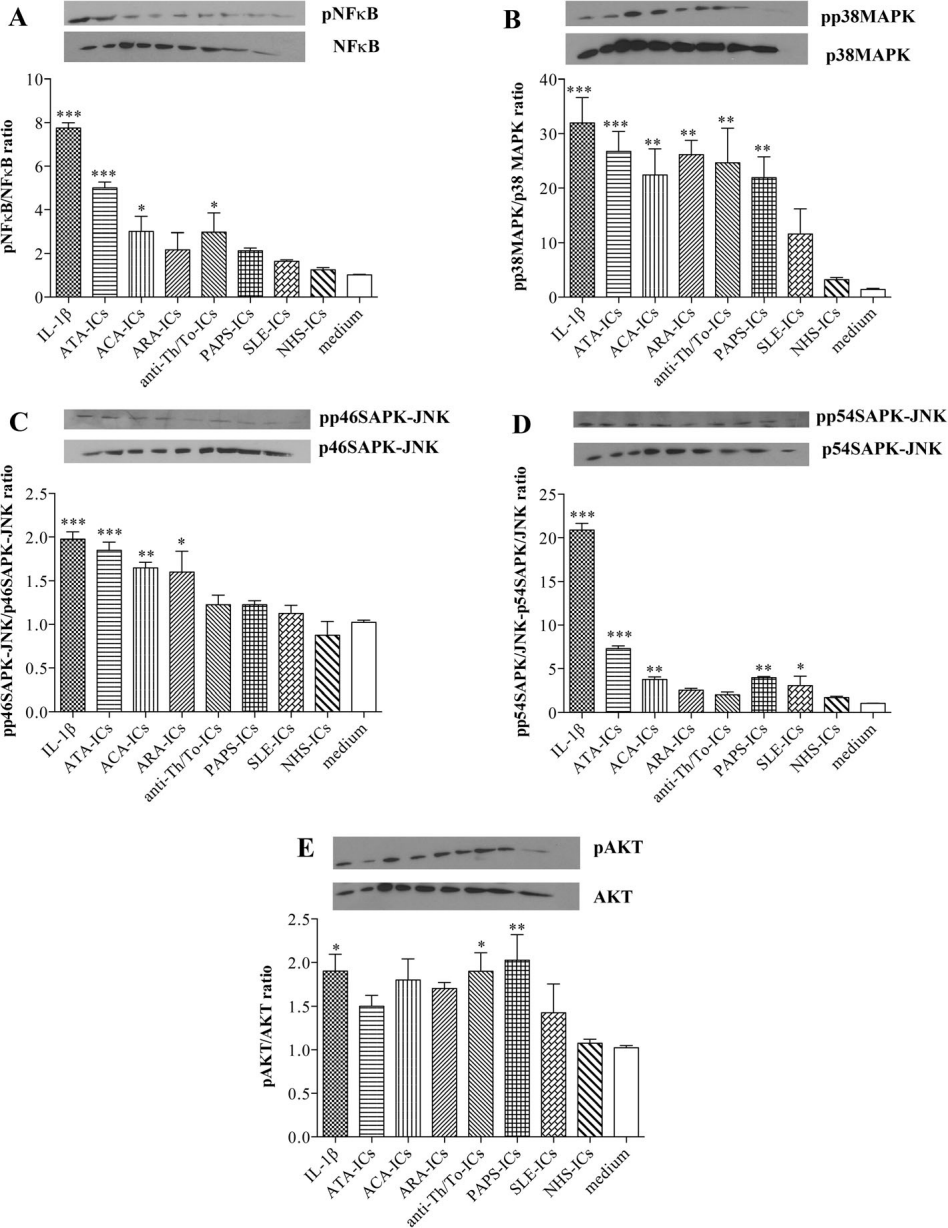
Intra-cellular signaling pathways in HUVECs stimulated with SSc-ICs, PAPS-ICs, SLE-ICs, or NHS-ICs. Endothelial cells were incubated with SSc-ICs, PAPS-ICs, SLE-ICs, or NHS-ICs (1:2 dilution). IL-1β (50 U/ml) was used as a positive control. Results are expressed as the ratio of phosphorylated to non-phosphorylated forms, evaluated using the ImageJ software. Western Blotting images are representative of a single experiment. pNFκB: phosphorylated NFκB; p38MAPK: phosphorylated p38MAPK; pp46SAPK-JNK: phosphorylated p46SAPK-JNK; pp54SAPK-JNK: phosphorylated p54SAPK-JNK; pAkt: phosphorylated Akt. Histograms represent mean + standard error of the mean (SEM). *p < 0.01; **p < 0.001; ***p < 0.0001 versus medium. **a** pNFκB/NFkB. IL-1β [7.75 ± 0.49] versus medium [1.02 ± 0.05] (*p* < 0.0001) versus NHS-ICs (*p* < 0.0001). ATA-ICs [5.00 ± 0.55] versus medium (*p* < 0.0001) and versus NHS-ICs [22.75 ± 7.59] (*p* < 0.0001). ACA-ICs [3.00 ± 1.39] versus medium (*p* < 0.01) versus NHS-ICs (*p* = N.S.). ARA-ICs [2.17 ± 1.55] versus medium (*p* = N.S.) versus NHS-ICs (*p* = N.S.). Anti-Th/To-ICs [2.98 ± 1.75] versus medium (*p* < 0.01) versus NHS-ICs (*p* = N.S.). PAPS-ICs [2.13 ± 0.26] versus medium (*p* = N.S.) versus NHS-ICs (*p* = N.S.). SLE-ICs [1.65 ± 0.13] versus medium (*p* = N.S.) versus NHS-ICs (*p* = N.S.). NHS-ICs [1.25 ± 0.21] versus medium (*p* = N.S.). **b** pp38/p38. IL-1β [31.98 ± 9.37] versus medium [1.4 ± 0.4] (*p* < 0.0001) versus NHS-ICs (*p* < 0.0001). ATA-ICs [26.75 ± 7.3] versus medium (*p* < 0.0001) and versus NHS-ICs [22.75 ± 7.59] (*p* < 0.001). ACA-ICs [22.43 ± 9.57] versus medium (*p* < 0.001) versus NHS-ICs (*p* < 0.01). ARA-ICs [26.15 ± 5.27] versus medium (*p* < 0.001) versus NHS-ICs (*p* < 0.001). Anti-Th/To-ICs [24.65 ± 12.77] versus medium (*p* < 0.001) versus NHS-ICs (*p* < 0.001). PAPS-ICs [21.93 ± 7.77] versus medium (*p* < 0.001) versus NHS-ICs (*p* < 0.01). SLE-ICs [11.63 ± 9.14] versus medium (*p* = N.S.) versus NHS-ICs (*p* = N.S.). NHS-ICs [3.2 ± 0.85] versus medium (*p* = N.S.). **c** pp46SAPK-JNK/p46SAPK-JNK. IL-1β [1.98 ± 0.17] versus medium [1.03 ± 0.05] (*p* < 0.0001) versus NHS-ICs (*p* < 0.0001). ATA-ICs [1.85 ± 0.19] versus medium (*p* < 0.0001) and versus NHS-ICs [22.75 ± 7.59] (*p* < 0.0001). ACA-ICs [1.65 ± 0.13] versus medium (*p* < 0.001) versus NHS-ICs (*p* < 0.0001). ARA-ICs [1.60 ± 0.48] versus medium (*p* < 0.01) versus NHS-ICs (*p* < 0.001). Anti-Th/To-ICs [1.23 ± 0.22] versus medium (*p* = N.S.) versus NHS-ICs (*p* = N.S.). PAPS-ICs [1.22 ± 0.09] versus medium (*p* = N.S.) versus NHS-ICs (*p* = N.S.). SLE-ICs [1.13 ± 0.19] versus medium (*p* = N.S.) versus NHS-ICs (*p* = N.S.). NHS-ICs [0.88 ± 0.32] versus medium (*p* = N.S.). **d** pp54SAPK-JNK/p54SAPK-JNK. IL-1β [20.88 ± 1.56] versus medium [1.05 ± 0.06] (*p* < 0.0001) versus NHS-ICs (*p* < 0.0001). ATA-ICs [7.30 ± 0.63] versus medium [21.75 ± 6.24] (*p* < 0.0001) and versus NHS-ICs [22.75 ± 7.59] (*p* < 0.0001). ACA-ICs [3.75 ± 0.59] versus medium (*p* < 0.001) versus NHS-ICs (*p* < 0.01). ARA-ICs [2.52 ± 0.41] versus medium (*p* = N.S.) versus NHS-ICs (*p* = N.S.). Anti-Th/To-ICs [2.02 ± 0.60] versus medium (*p* = N.S.) versus NHS-ICs (*p* = N.S.). PAPS-ICs [3.98 ± 0.24] versus medium (*p* < 0.001) versus NHS-ICs (*p* < 0.01). SLE-ICs [3.05 ± 2.17] versus medium (*p* < 0.01) versus NHS-ICs (*p* = N.S.). NHS-ICs [1.73 ± 0.28] versus medium (*p* = N.S.). **e** pAkt/Akt. IL-1β [1.90 ± 0.39] versus medium [1.02 ± 0.05] (*p* < 0.01) versus NHS-ICs (*p* < 0.01). ATA-ICs [1.50 ± 0.24] versus medium [21.75 ± 6.24] (*p* = N.S.) and versus NHS-ICs [22.75 ± 7.59] (*p* = N.S.). ACA-ICs [1.80 ± 0.48] versus medium (*p* = N.S.) versus NHS-ICs (*p* = N.S.). ARA-ICs [1.70 ± 0.14] versus medium (*p* = N.S.) versus NHS-ICs (*p* = N.S.). Anti-Th/To-ICs [1.90 ± 0.42] versus medium (*p* < 0.01) versus NHS-ICs (*p* < 0.01). PAPS-ICs [2.02 ± 0.59] versus medium (*p* < 0.001) versus NHS-ICs (*p* < 0.01). SLE-ICs [1.42 ± 0.65] versus medium (*p* = N.S.) versus NHS-ICs (*p* = N.S.). NHS-ICs [1.08 ± 0.10] versus medium (*p* = N.S)

ATA-ICs, ACA-ICs, anti-Th/To-ICs, PAPS-ICs, and IL-1β significantly activated NFκB compared to the medium whereas ARA-ICs, SLE-ICs, and NHS-ICs did not elicit NFκB phosphorylation (Fig. 4a). All SSc-ICs, in particular, ATA-ICs and anti-Th/To-ICs, induced a significant increased phosphorylation rate of p38MAPK compared to the medium. PAPS-ICs and IL-1β also activated p38MAPK, whereas SLE-ICs and NHS-ICs did not exert any effect on the activation rate of p38MAPK (Fig. 4b).

ATA-ICs, ACA-ICs, ARA-ICs, and IL-1β drove a significant increased phosphorylation rate of p46SAPK-JNK. Anti-Th/To-ICs and NHS-ICs, as well as PAPS-ICs and SLE-ICs, did not exert any effect on the phosphorylation rate of p46SAPK-JNK (Fig. 4c). ATA-ICs, ACA-ICs, PAPS-ICs, and IL-1β yielded a significant increase in the p54SAPK-JNK phosphorylation rate. Conversely, ARA-ICs, anti-Th/To-ICs, SLE-ICs, and NHS-ICs did not affect the activation rate of p54SAPK-JNK (Fig. 4d). All SSc-ICs, PAPS-ICs, and IL-1β resulted in a significant activation of Akt compared to medium; SLE-ICs and NHS-ICs did not elicit any effect on Akt phosphorylation rate (Fig. 4e).

**Fig. 5:**
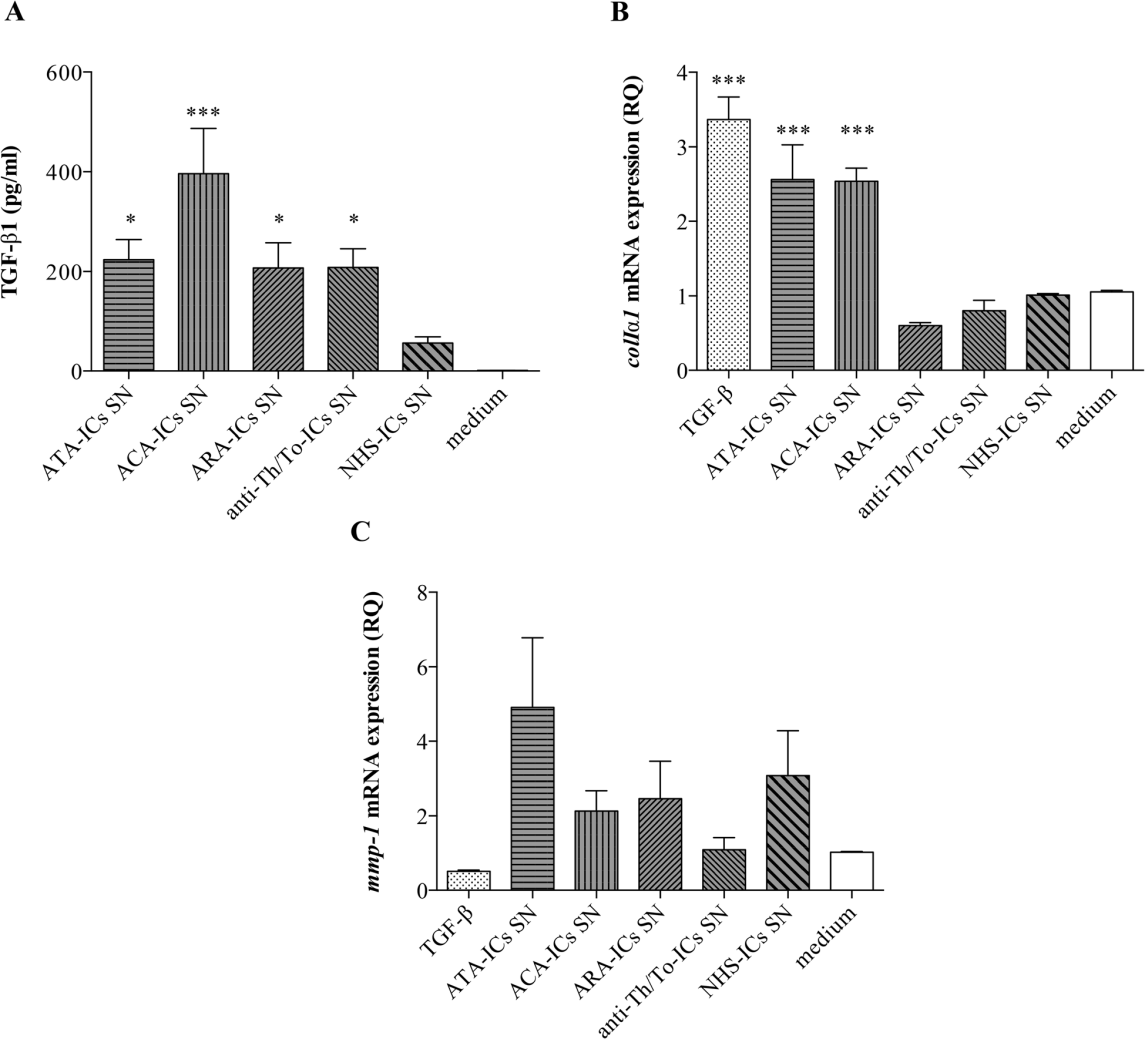
TGF-β1 secretion and *colIα1* and *mmp-1* mRNA expression in fibroblasts stimulated with supernatants from HUVECs incubated with SSc-ICs or NHS-ICs. Fibroblasts were exposed to supernatants from HUVECs incubated with SSc-ICs or NHS-ICs (1:2 dilution). TGF-β1 (10 ng/ml) was used as a positive control for collagen and *mmp-1* synthesis. Histograms represent mean + standard error of the mean (SEM). *p < 0.01; ***p < 0.0001 versus medium. **a** TGF-β1 (pg/ml) (mean ± SD). ATA-ICs [223.2 ± 90.60] versus medium [1.08 ± 0.22] (*p* < 0.01) and versus NHS-ICs [56.25 ± 24.92] (*p* = N.S.). ACA-ICs [396.20 ± 203.90] versus medium (*p* < 0.0001) versus NHS-ICs (*p* < 0.0001). ARA-ICs [207.40 ± 111.70] versus medium (*p* < 0.01) versus NHS-ICs (*p* = N.S.). Anti-Th/To-ICs [208.40 ± 97.70] versus medium (*p* < 0.01) versus NHS-ICs (*p* = N.S.). NHS-ICs [56.25 ± 24.92] versus medium (*p* = N.S.). **b** *colIα1* (RQ) [mean ± SD]. TGF-β [3.37 ± 0.42] versus medium [1.05 ± 0.04] (*p* < 0.0001) versus NHS-ICs (*p* < 0.0001). ATA-ICs [2.56 ± 0.93] versus medium (*p* < 0.0001) and versus NHS-ICs [1 ± 0.5] (*p* < 0.0001). ACA-ICs [2.54 ± 0.35] versus medium (*p* < 0.0001) versus NHS-ICs (*p* < 0.0001). ARA-ICs [0.60 ± 0.08] versus medium (*p* = N.S.) versus NHS-ICs (*p* = N.S.). Anti-Th/To-ICs [0.80 ± 0.28] versus medium (*p* = N.S.) versus NHS-ICs (*p* = N.S.). NHS-ICs [1 ± 0.4] versus medium (*p* = N.S.). **c** *mmp-1* (RQ) [mean ± SD]. TGF-β [0.51 ± 0.04] versus medium [1.02 ± 0.04] (*p* = N.S.) versus NHS-ICs (*p* = N.S.). ATA-ICs [4.91 ± 3.74] versus medium (*p* = N.S.) and versus NHS-ICs [3.08 ± 2.40] (*p* = N.S.). ACA-ICs [0.13 ± 1.09] versus medium (*p* = N.S.) versus NHS-ICs (*p* = N.S.). ARA-ICs [2.46 ± 2.0] versus medium (*p* = N.S.) versus NHS-ICs (*p* = N.S.). Anti-Th/To-ICs [1.09 ± 0.65] versus medium (*p* = N.S.) versus NHS-ICs (*p* = N.S.). NHS-ICs [3.08 ± 2.40] versus medium (*p* = N.S)

### TGF-β1 secretion, *colIα1*, and *mmp-1* mRNA expression in fibroblasts stimulated with supernatants from endothelial cells treated with immune complexes

Supernatants from HUVECs treated with all SSc-ICs induced a significant increase in TGF-β1 secretion compared to the medium in skin fibroblasts, whereas NHS-ICs did not (Fig. 5a). A significant upregulation of *colIα1* was observed with ATA-ICs and ACA-ICs. ARA-ICs, anti-Th/To-ICs, and NHS-ICs did not elicit a significant modulation of *colIα1* expression. TGF-β stimulation resulted in a significant rise of *colIα1* levels in fibroblasts (Fig. 5b). SSc-ICs and NHS-ICs as well as TGF-β did not affect the *mmp-1* expression in fibroblasts (Fig. 5c).

### α-SMA and IL-6 protein expression in fibroblasts stimulated with supernatants from endothelial cells treated with immune complexes

**Fig. 6:**
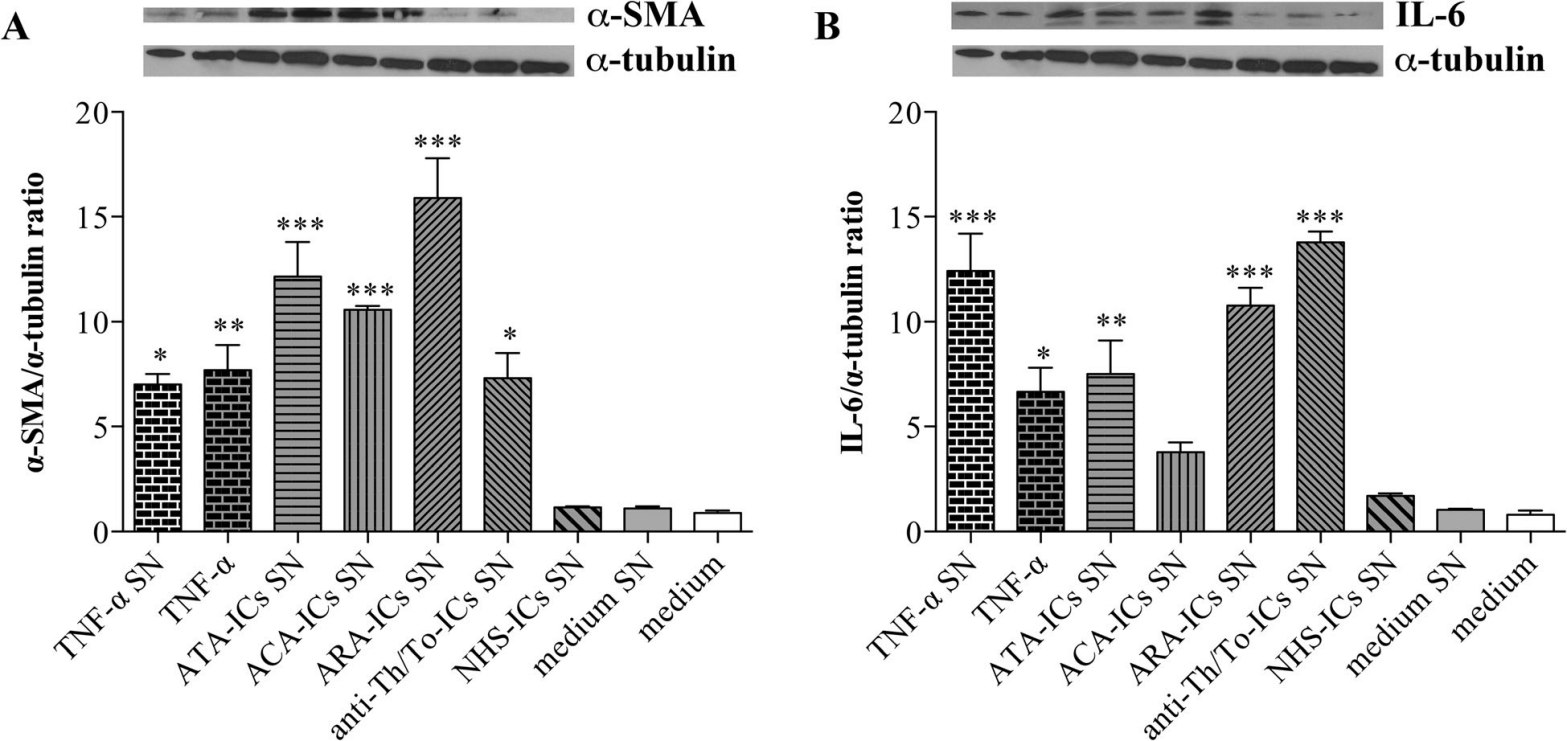
α-SMA and IL-6 protein expression in fibroblasts stimulated with supernatants from HUVECs incubated with SSc-ICs and NHS-ICs. Fibroblasts were exposed to supernatants from HUVECs incubated with TNF-α (10 ng/ml), SSc-ICs, or NHS-ICs (1:2 dilution). TNF-α (10 ng/ml) was used as a positive control. SN, supernatants. Histograms represent mean + standard error of the mean (SEM). *p < 0.01; ***p < 0.0001 versus medium. **a** α-SMA. TNF-α SN [7.0 ± 0.71] versus medium SN [1.1 ± 0.14] (*p* < 0.01) versus NHS-ICs SN (*p* < 0.01). TNF-α [7.70 ± 1.70] versus medium [0.9 ± 0.15] (*p* < 0.001). ATA-ICs SN [12.15 ± 2.33] versus medium SN (*p* < 0.0001) and versus NHS-ICs SN [1.15 ± 0.07] (*p* < 0.0001). ACA-ICs SN [10.58 ± 0.25] versus medium SN (*p* < 0.0001) versus NHS-ICs SN (*p* < 0.0001). ARA-ICs SN [15.90 ± 2.70] versus medium SN (*p* < 0.0001) versus NHS-ICs SN (*p* < 0.0001). Anti-Th/To-ICs SN [7.30 ± 1.69] versus medium SN (*p* < 0.01) versus NHS-ICs SN (*p* < 0.01). NHS-ICs SN [1.15 ± 0.07] versus medium SN (*p* = N.S.). **b** IL-6. TNF-α SN [12.41 ± 2.54] versus medium SN [1.05 ± 0.07] (*p* < 0.0001) versus NHS-ICs SN (*p* < 0.0001). TNF-α [6.65 ± 1.63] versus medium [0.08 ± 0.28] (*p* < 0.01). ATA-ICs SN [7.50 ± 2.26] versus medium SN (*p* < 0.001) and versus NHS-ICs SN [1.71 ± 0.17] (*p* < 0.01). ACA-ICs SN [3.77 ± 0.66] versus medium SN (*p* = N.S.) versus NHS-ICs SN (*p* = N.S.). ARA-ICs SN [10.77 ± 1.22] versus medium SN (*p* < 0.0001) versus NHS-ICs SN (*p* < 0.0001). Anti-Th/To-ICs SN [13.78 ± 0.73] versus medium SN (*p* < 0.0001) versus NHS-ICs SN(*p* < 0.0001). NHS-ICs SN [1.71 ± 0.17] versus medium SN (*p* = N.S)

Supernatants from HUVECs treated with all SSc-ICs induced a significant increase in α-SMA protein expression compared to the medium in skin fibroblasts, whereas NHS-ICs did not. A significant upregulation of α-SMA was observed even when fibroblasts were treated with TNF-α and with supernatants from HUVECs treated with TNF-α (Fig. 6a). Supernatants from HUVECs treated with all SSc-ICs but ACA-ICs significantly upregulated IL-6 protein expression compared to the medium in skin fibroblasts, whereas NHS-ICs did not. A significant upregulation of IL-6 was observed even when fibroblasts were treated with TNF-α and with supernatants from HUVECs treated with TNF-α (Fig. 6b).

### Pre-treatment with IL-6 and TGF-β inhibitors in fibroblasts stimulated with supernatants from endothelial cells treated with ATA-ICs

Pre-treatment with both IL-6 and TGF-β inhibitors significantly reduced the mRNA levels of *et-1* in fibroblasts stimulated with supernatants from HUVECs incubated with ATA-ICs compared to not pre-treated cells. In particular, pre-treatment with IL-6 inhibitor resulted in an 85% reduction of *et-1* mRNA expression levels whereas the pre-incubation with TGF-β blocking antibody led to a 77% downregulation of *et-1* mRNA expression.

## Discussion

This in vitro work demonstrates for the first time that scleroderma-specific autoantibodies embedded in ICs induce a pro-inflammatory and pro-fibrotic phenotype even at the endothelial level. The hereby presented findings in endothelial cells expand our previous observations on the contribution of SSc-ICs to disease pathogenesis, which were raised in healthy skin fibroblasts. In particular, endothelial incubation with SSc-ICs modulates several molecules involved in the three cardinal scleroderma pathophysiologic processes: vascular dysfunction (ET-1 and IL-8), inflammation (ICAM-1, IL-6), and fibrosis (TGF-β1). Conversely, no modulation of type I IFNs and *colIα1* emerges, possibly due to the fact that endothelial cells are not master producers of these mediators. The data presented in this work acquire particular relevance given the fundamental role of endothelial cells in the pathogenesis of SSc. Indeed, endothelial damage is regarded as the very first event in the disease course, as suggested by the evidence that Raynaud’s phenomenon is almost invariably presenting clinical manifestation in scleroderma [3]. Once activated, endothelial cells contribute to disease pathogenesis mediating the fibroproliferative vasculopathy characteristic of SSc: the unbalanced production of vasoactive mediators results in vasoconstriction; the increased expression of adhesion molecules by damaged endothelial surface promotes leukocyte trans-endothelial migration, activation, and accumulation; endothelial cells transdifferentiate into myofibroblasts gaining mesenchymal cell markers. The above-cited events culminate in the intima and media proliferation and vessel occlusion ultimately leading to tissue hypoxia, which further promotes cell injury and fibroblast activation [14, 22]. Several potential insults to the endothelium, for instance, viral infections, cold exposure, or antibodies against endothelial cells, have been proposed as the initial trigger in SSc etiopathogenesis, without conclusive results. Our data were raised using healthy endothelial cells, thus suggesting that scleroderma-specific antibodies embedded in ICs might contribute to endothelial damage early on the disease course. As a support, positivity for scleroderma antibodies and microvasculopathy signs detected by capillaroscopy are consistently identified as the main predictors of evolution into SSc among patients with Raynaud’s phenomenon [23]. The damage of endothelial cells induced by SSc-ICs might even precede fibroblast activation. Indeed, our data provide evidence for a direct interaction between endothelial cells and fibroblasts: upon stimulation with medium from endothelial cell culture treated with SSc-ICs, fibroblasts synthetize a higher amount of *colIα1*, *tgf-β1*, α-SMA, and IL-6. In agreement with previous studies [24–27], these findings strongly suggest that endothelial cells can impact the function of fibroblasts inducing a pro-fibrotic phenotype and that such effect on fibroblasts might be mediated by both IL-6 and TGF-β synthetized by endothelial cells in response to SSc-IC stimulation, as documented by the high inhibition rates in *et-1* mRNA levels.

The potential pathogenic relevance of antibodies in scleroderma is also suggested by the accumulation of B lymphocytes at sites of diseases, such as around the small vessels in affected skin and in the alveolar interstitium in patients with lung involvement. In SSc, autoreactive B cells, which display high affinity for the antigens, escape censure due to defective B lymphocyte selection and regulation, with increased production of IL-6 and IL-8 and decreased synthesis of IL-10 [28]. Further support comes from the evidence of the effectiveness of anti-B cell therapeutic approach: in vivo, the inhibition of CD19 has been shown not only to abrogate autoantibody production but also to ameliorate skin fibrosis; SSc patients, particularly those ATA-positive, benefit from rituximab treatment [29]. The postulate of the pathogenic relevance of functional autoantibodies in SSc pathogenesis has always been appealing for the scleroderma research community, possibly due to the deleterious effects on cells of treatment with sera from patients [30]. Back in the 1970s, anti-endothelial antibodies were reported in 30% of SSc patients [31]; in early 2000, the attention shifted to anti-fibroblast antibodies [32]. Even recently, the research efforts have been focused on additional newly discovered functional autoantibodies reacting against cell surface receptors. This is the case of antibodies against angiotensin II type I receptor (AT1R), endothelin-1 type A receptor (ETAR), and platelet-derived growth factor receptor (PDGFRα) [33]. Support to the pathogenic potential of these functional autoantibodies has been raised both in vitro and in vivo, being however suggestive of a cell characteristic activation of pathogenic pathways that might account for specific disease manifestations: vascular damage for anti-AT1R and anti-ETAR, and fibrosis for anti-PDGFRα antibodies. In addition, these autoantibodies can be detected in a minority of patients’ sera and display a much lower specificity compared to SSc-specific antibodies [33]. Our previous work documenting the pathogenicity of SSc-ICs on skin fibroblasts has energized the research on the functional role of SSc-specific autoantibodies. In particular, it has been very recently documented that sera containing ATA and ACA as well as polyclonal IgG antibodies targeting DNA topoisomerase I and centromeric protein B affect the viability and apoptosis rate of healthy skin fibroblasts. The same treatments upregulated pro-fibrotic molecules as α-SMA, colIα1, and transgelin, assessed both as mRNA expression and as protein levels at immunochemistry [34]. To overcome criticisms advocating the nuclear localization of both antigens, the authors claimed that, according to previous studies, DNA topoisomerase I and centromeric protein B can be released from damaged endothelial cells [35, 36]. Our model bypasses the issue of the nuclear localization of antigens since it envisages the interaction of SSc-ICs with target cells to be mediated by nucleic acids embedded in SSc-ICs. Indeed, scleroderma-specific antibodies are directed against a limited set of autoantigens, comprising DNA, RNA, or acid nucleic-binding proteins. In our previous work, we have raised evidence for a significant enrichment in nucleic acids of SSc-ICs, which has been recently confirmed by an independent group [37]. These fragments might be exogenous or endogenous in nature, originating from microbes or self-cells. Interestingly, neutrophil elastase-expressing cells (granulocytes and activated macrophages), all extensively found to infiltrate scleroderma skin, have been recently identified as the main source of self-DNA [37].

According to the present data as well as to our previous study, SSc-ICs might signal in target cells through the interaction of nucleic acids with TLRs. The present work suggests that TLRs mediating cell response to SSc-ICs reside in the cell membrane and not in the intra-cellular compartments. Indeed, to characterize the cell localization of TLRs interacting with SSc-ICs, endothelial cells have been pre-incubated with bafilomycin, an inhibitor of the vacuolar-type H+-ATPase, which prevents the activation of intra-cellular TLRs by interfering with endosomal acidification. We evince that pre-treatment with bafilomycin does not affect at any rate the upregulation of *il-6* and *et-1* induced by ATA-ICs, ACA-ICs, and anti-Th/To. Conversely, a partial modulation of both genes has been observed upon treatment with ARA-ICs. The negligible involvement of endosomal TLR3 and TLR9 in endothelial cell response to SSc-ICs is in agreement with the lack of expression of FcγRs in HUVECs. In dendritic cells and B lymphocytes, SSc autoantibodies were previously shown to be internalized via FcγRs [38]; however, this hypothesis cannot be translated to the endothelium. Indeed, in agreement with other authors, we detect a minimal expression of CD64 while CD32 and CD16 were not detectable [39–41]. Since CD32 displays a high affinity for IgG ICs and is deputed not only to IC uptake but also to intracellular signal transduction, these data suggest that FcγRs do not mediate endothelial cell response to SSc-ICs.

In fibroblasts, stimulation with ICs culminated in the upregulation of *tlr2*, *tlr3*, *tlr4*, and, to a lower extent, *tlr9* [13]. In the present work, we observe some differences in TLR modulation in endothelial cells compared to previous data on fibroblasts, suggesting a cell-specific response to treatment with SSc-ICs. Indeed, ATA-ICs and ACA-ICs engage *tlr2*, *tlr3*, and *tlr4*, whereas *tlr9* is recruited upon anti-Th/To-ICs. This hypothesis might be novel for SSc but has been accepted long ago in other systemic autoimmune diseases. It has been indeed demonstrated, both in in vitro and in vivo studies, that DNA- and RNA-containing ICs from SLE patients stimulate plasmacytoid dendritic cells and B cells to secrete IFN-α through TLR9 and TLR7 via the nucleic acid residues [42–46].

In addition, an increasing burden of evidence points towards a prominent and deleterious role in SSc etiopathogenesis of chronically activated TLRs. There are solid and growing data in support of TLR involvement in SSc: ex vivo data document overexpression of TLRs in lesional tissues from patients; in vitro experiments highlight the overexpression of pro-fibrotic markers upon TLR activation; in vivo studies in different animal models evince the role of TLRs in mediating tissue fibrosis; genetic studies reveal the association of SSc with polymorphisms in *tlr* genes [47]. In addition, SSc patients display increased levels of endogenous ligands of TLR2 (serum amyloid A and S100A7), TLR4 (tenascin C, fibronectin, high-mobility group protein B1 [HMGB1], and heat shock protein 90). Most recently, ICs composed of DNA and CXCL4 (chemokine (C-X-C motif) ligand 4), a cytokine overexpressed in SSc skin, have been identified as novel TLR ligands in SSc, amplifying TLR9 activation and IFN-α production in plasmacytoid dendritic cells [37].

The cell specificity of the response to SSc-ICs is further suggested by the differential pattern of activation of intra-cellular mediators in endothelial cells as compared to fibroblasts. At the endothelial level, ATA-ICs and ACA-ICs engage NFκB, p38MAPK, p46SAPK-JNK, p54SAPK-JNK, and Akt. Conversely, a selective activation is registered for ARA-ICs and anti-Th/To-ICs: the first results in the recruitment of p38MAPK, p46SAPK-JNK, and Akt, while the latter promotes the activation of p38MAPK and Akt. As expected, stimulation with PAPS-ICs yields the activation of NFkB, p38MAPK, p54SAPK-JNK, and Akt whereas SLE-ICs do not affect the activation rate of any intracellular mediator. Such a striking difference might be explained by the etiopathogenic features of the two diseases. Even though in both conditions autoantibodies have been shown to exert not only a diagnostic but also a pathogenic role, aPL display a particular tropism for the endothelium, which provides their main cellular target [9]. Furthermore, in the present work, we confirm our previous finding that ICs from SLE and PAPS patients elicit a differential modulation of study mediators compared to SSc-ICs [13], without affecting molecules directly involved in fibrogenesis such as TGF-β1.

SSc-ICs might contribute to scleroderma etiopathogenesis being responsible for chronic and aberrant activation of TLRs on several cells involved in SSc. Thus, our hypothesis envisages a direct interaction of ICs with TLRs and does not postulate a type III hypersensitivity mechanism, in which small-sized ICs are ineffectively cleared from circulation, deposit in tissues where activate complement leading to a prominent inflammatory response [48]. Indeed, histological examination of scleroderma skin samples reveals lymphocytic and plasma cell infiltrate in the perivascular mid-lower dermis, subcutis, and subcutaneous fat but Ig staining is typically negative; there is no complement deposition in tissues, and serum complement is not consumed [49].

Our data document a characteristic pattern of modulation of cell functionality for each SSc-IC preparation; thus, it would be surely intriguing to hypothesize that scleroderma-associated antigenic specificities might account for the clinical phenotype we observe in patients with a given autoantibody profile. Indeed, the stimulation of both HUVECs and, in turn, of fibroblasts with ATA-ICs results in a marked pro-inflammatory and pro-fibrotic phenotype, a finding that fits well with clinical features of patients carrying ATA. Similarly, ACA-ICs elicit a strong response in HUVECs, consistently with the prevalent vascular involvement that characterizes clinical presentation in patients. Even though our work clearly supports the pathogenic potential of SSc-ICs in both endothelial cells and fibroblasts, we still believe that a definitive conclusion on the differential pathogenic potential of SSc-ICs should be drawn cautiously. Indeed, clinical manifestations arise from a close interplay between several cells in a tissue-specific milieu; our in vitro model might be over-simplistic, not allowing to adequately reproduce the complexity of scleroderma pathogenesis. Such partial insight into disease etiopathogenesis provides one of the limitations that flaw our study. To overcome this critical issue, we are implementing a dynamic in vitro model that envisages multiple bioreactors for 3D cell cultures interconnected by a dynamic flow in order to mimic the cell crosstalk that characterizes SSc pathogenesis in humans [50]. Another major limitation of this study relates to the scarce number of samples for each autoantibody specificity; unfortunately, the recruitment of a broader cohort of patients is prevented by the relative rarity of certain antibodies (e.g., ARA and anti-Th/To) in the Italian SSc population [51]. We acknowledge that the number of disease controls is low, due to the technical requirement of fresh PEG preparations for in vitro cellular studies. Despite the limited number of samples, the robustness of the conclusions presented in this study, which provides the proof-of-concept of IC deleterious effects on the endothelium, is preserved.

## Conclusion

As a whole, the present work provides novel insights into the pathogenicity of SSc-ICs, yielding a demonstration of the detrimental effects of preparations containing scleroderma autoantibodies on the endothelium. SSc-ICs might be located at a cross-road in scleroderma pathogenesis: future studies should focus on how ICs contribute to the activation of several cells involved in scleroderma pathogenesis. Besides fibroblasts and endothelial cells, SSc-ICs might interact with several other cellular players involved in scleroderma pathogenesis: adipocytes, T cells, macrophages, and plasmacytoid dendritic cells. Insights into the SSc-IC pathogenicity might have therapeutic implications, identifying TLRs as candidate pharmacological targets in the initiation phase of the disease, before the onset of overt fibrosis. TLR signaling can be disrupted in multiple ways: blocking TLRs with an antibody, enhancing negative regulators of TLR signaling, and/or modifying the epigenetic regulators of TLR signaling. In vivo data have already been raised, since TLR4 and TLR9 antagonists prevented and reversed organ fibrosis in distinct preclinical disease models, highlighting the potential therapeutic benefit of selectively blocking TLR4 activity [47, 52, 53]. Hydroxychloroquine, an anti-malarial whose TLR-modulating properties are well established, has been recently shown to ameliorate serum markers of endothelial injury [54]. This future might not be so far away: monospecific TLR7 and TLR9 antagonists are currently under investigation for the treatment of dermatomyositis and plaque psoriasis [52].

## Data Availability

The datasets supporting the conclusions of this article can be made available upon request.

## Abbreviations

SSc: Systemic sclerosis
ECM: Extra-cellular matrix
PAH: Pulmonary arterial hypertension
SRC: Scleroderma renal crisis
GAVE: Gastric antral vascular ectasia
ATA: Anti-DNA topoisomerase I antibodies
ILD: Interstitial lung disease
ACA: Anti-centromeric protein antibodies
ARA: Anti-RNA polymerase III antibodies
anti-Th/To: Anti-Th/To antibodies
SLE: Systemic lupus erythematosus
PAPS: Primary anti-phospholipid syndrome
ICs: Immune complexes
ANA: Anti-nuclear antibodies
RNP: Ribonucleoprotein
NHS: Normal healthy subjects
HUVECs: Human umbilical vein endothelial cells
FBS: Fetal bovine serum
colIα1: CollagenIα1
MMP: Matrix metalloproteinase
TGF: Transforming growth factor
SMA: Smooth muscle actin
PEG: Polyethylene glycol
RPMI: Roswell Park Memorial Institute
ICAM-1: Inter-cellular adhesion molecule
OD: Optical density
LAL: Limulus amoebocyte lysate
ELISA: Enzyme-linked immunosorbent assay
HBSS: Hank’s Balanced Salt Solution
IL: Interleukin
IFN: Interferon
et-1: Endothelin-1
NFκB: Nuclear factor κ B
pNFκB: Phosphorylated nuclear factor κ B
p38MAPK: p38 mitogen-activated kinase
pp38MAPK: Phosphorylated p38MAPK
JNK: JUN N terminal kinase
Akt: RAC-α serine/threonine-protein kinase
P/T: PBS/0.05% Tween 20
SD: Standard deviation
AT1R: Angiotensin II type I receptor
ETAR: Endothelin-1 type A receptor
PDGFR: Platelet-derived growth factor receptor
DAMPs: Damage-associated molecular patterns
HMGB1: High-mobility group protein B1
FcγR: Fcγ receptor
CXCL4: Chemokine (C-X-C motif) ligand 4
